# Sleep, sedentary behavior, and physical activity in Brazilian adolescents: Achievement recommendations and BMI associations through compositional data analysis

**DOI:** 10.1371/journal.pone.0266926

**Published:** 2022-04-11

**Authors:** Sabrina Fontes Domingues, Cristiano Diniz da Silva, Fernanda Rocha Faria, Helton de Sá Souza, Paulo Roberto dos Santos Amorim

**Affiliations:** 1 Department of Physical Education, Federal University of Viçosa, Viçosa, Minas Gerais, Brazil; 2 Department of Physical Education, Federal University of Juiz de Fora—Advanced Campus Governador Valadares, Governador Valadares, Minas Gerais, Brazil; 3 Federal Institute of Education, Science and Technology of Triângulo Mineiro, Ituiutaba, Minas Gerais, Brazil; IRCCS Istituto Delle Scienze Neurologiche di Bologna, ITALY

## Abstract

Physical activity, sedentary behavior (SB), and sleep are habitual human behaviors (HHB) which are modifiable throughout the different life phases. Therefore, it is necessary to analyze how the time distribution throughout the day among HHB may be associated with body mass index (BMI). These results could provide inferences which can guide interventions that trigger changes in adolescent behaviors in favor of their health. The objective of this study was to verify the proportion of adolescents who meet the recommendation of sleep, moderate to vigorous physical activity (MVPA), and screen time (ST); to analyze the associations between HHB and BMI, and to determine possible changes in BMI associated with time reallocation between different HHB. Daily HHB recommendations (yes/no) were analyzed by frequency distribution. Compositional data analyses were used to examine the association between HHB and the BMI z-score (*z*BMI) with covariates (sex, age, and socioeconomic status). Compositional isotemporal substitution models estimated the change in *z*BMI associated with HHB reallocations from 15 to 120 minutes. A total of 185 adolescents were included (15 to 18 years, 50.8% boys). Thus, total sleep time, SB, light physical activity (LPA) and MVPA were measured by 24-hour accelerometry for seven consecutive days. ST, demographic characteristics, and socioeconomic status were assessed using a questionnaire. Sleep, MVPA, and ST recommendations were achieved by 32.97%, 8.10%, and 1.08% of the sample, respectively. No adolescent was able to achieve all of the daily recommendations. Age was significantly and positively associated with zBMI (*p*<0.001). Simply replacing 75, 90, and 120 minutes of MVPA by LPA led to an estimated significant increase in *zBMI* (*95CI% z-value*, *0*.*01 to 1*.*49*). The HHB relocation estimates in 24h did not show positive effects on *z*BMI, nor did it increase the time engaged in MVPA, which may raise the hypothesis that other parameters related to obesity and their related interactions need to be better understood.

## Introduction

“Movement behaviors” have been cited in the literature [1, 2] as encompassing physical activity (PA), sedentary behavior (SB) and sleep. They have been influenced by various interdependent factors regarding the time-lapse over a day. Therefore, they are modifiable [3] throughout the different life phases. The term habitual human behaviors (HHB) was adopted due to possible concepts counterpoints, considering the definition of sleep as a behavioral, reversible, and cyclic state characterized by relative immobility and increased threshold of responses to external stimuli [4, 5].

Adolescence is a period of life characterized by biopsychosocial, cognitive, and behavioral changes that affect the entire life of subjects [6, 7]. Among these changes, it is possible to observe a marked change in the sleep-wake cycle, with a predisposition to a later sleep-wake cycle causing a delay in the circadian rhythm by a reduction in the quality and total sleep time [8]. It is worth mentioning that sleep disorders can negatively interfere with cognition [9], mood [10], and metabolism [11], in addition to the endocrine, immune and cardiovascular systems [12].

Certainly, all of the aforementioned alterations may favor an increase in sedentary behavior [13] typically observed in adolescence. These alterations may increase the risk of cardiovascular and all-cause mortality, as well as favor the incidence of cardiovascular disease and type II diabetes [14]. The literature has shown that most adolescents do not meet daily public health recommendations, which cover a minimum of 60 minutes of moderate to vigorous PA (MVPA) practice [1, 15–17], from eight to 10 hours of sleep [1, 18, 19], and a maximum of two hours of screen time (ST) [1, 20], which can all impact this population’s body mass index (BMI).

In addition, it is well-known that obesity in adolescence can negatively affect several health components [21] and may persist in adulthood, increasing the risk of developing chronic diseases throughout life [22] and favoring early mortality. Therefore, it is necessary to analyze how the time distribution throughout the day among HHB may be associated with BMI to search for inferences which can guide interventions that trigger changes in adolescent behaviors in favor of their health.

Behaviors represent mutually exclusive components of daily time use [23, 24]. Therefore, they present data of a compositional nature [24–26] in which their constant sum results in 24 hours [27, 28]. In this sense, several studies have used compositional techniques to assess the relationships with health [22, 24, 25, 27–33], and non-conventional multivariate analysis methods are required to consider co-dependence and collinearity [25, 26].

The use of a compositional isotemporal substitution model also offers the possibility of quantifying the time spent in each behavior and relocating fixed periods between the 24-hour behavior [28], thereby allowing to verify associations and joint theoretical predictions between the 24-hour behavior and health [34]. However, a recent literature review with 51 studies [35] indicated that only one study was conducted with adolescents verifying behaviors and BMI. In addition to this gap, no study has evaluated possible BMI changes when relocating fixed periods between HHB evaluated by 24-hour accelerometry in a sample of Brazilian adolescents.

Given the above, this study aims to: i) verify the proportion of adolescents who comply with the behavioral recommendations of sleep, MVPA, and ST and their combinations; ii) analyze the associations between HHB and BMI through compositional analysis; and iii) estimate the theoretical effects of isotemporal substitution between these HHB and BMI.

## Materials and methods

### Study design and participants

This is an observational cross-sectional study composed of adolescent students of both sexes from the Federal Institute of the Triângulo Mineiro (IFTM), a full-time public school located in Ituiutaba city, Minas Gerais, Brazil. According to the Brazilian Institute of Geography and Statistics [36], Ituiutaba city comprised a population of 105,818 inhabitants in 2021, and the Human Development Index was 0.739. The study was conducted between March and September 2018. Adolescents under 18 years of age signed a free and informed consent form (ICF) to participate in the study, while their parents or legal guardians signed the free and informed assent form (IAF). Adolescents aged 18 years or older only received the ICF, which they had to sign. All procedures met the guidelines of the Declaration of Helsinki, being approved by the Committee of Ethics in Research involving human beings of the Federal University of Viçosa (Presentation Certificate of Ethical Appreciation—CAAE: 74104217.3.0000.5153, report no: 2.313.053). The dataset analyzed during the current study are available in S1 File. This study is an integral part of an umbrella project that evaluates movement behavior and health outcomes in adolescents [37]. The sample size was calculated by the EpiInfo version 7.2.2.16 software program (Georgia, USA) using a formula for cross-sectional studies.

The population size was established at 471 (number of adolescents enrolled in technical courses integrated to the IFTM high school, Ituiutaba Campus). A prevalence of 26% overweight and obesity in Brazilian adolescents aged 15 to 17 years was assumed for this study [38], as well as an acceptable error of 5%, a confidence level of 95%, and a design effect of 1.0. Thus, a minimum sample of 185 adolescents was found from these configurations.

The volunteers were selected by simple random sampling, respecting sex proportionality and the high school grade. The result was disregarded in cases where the student did not agree to participate in the study, and a new selection was performed.

Students regularly enrolled in the institute’s high school, between 15 and 18 years of age and not participating in weight loss programs, were included. Girls who had menarche for at least of 1 year, and boys who had axillary hair were included. Pregnant adolescents, those who had any mental or physical disabilities (temporary or permanent), who used diuretics or antihypertensives, those who had not signed the ICF or IAF forms or those who did not use the accelerometer for at least three nights (two weekdays and one weekend day) were excluded.

Details of data collection procedures can be found in a previous publication by our research group [37].

### Data collection procedures

#### Anthropometry

Bodyweight was individually measured using a digital scale (Plenna^**®**^, model Ice HON-00823, São Paulo, Brazil) and height was measured by a portable stadiometer (Sanny Medical^**®**^, model ES-2040, São Paulo, Brazil) according to Lohman, Roche, and Martorell [39] in a specific room with a controlled environment. In addition, BMI was classified by z-score according to sex and age by the World Health Organization [40].

#### Screen time

The adolescents were instructed to consider all types of screens when asked: “On a normal day, how many hours do you spend in front of an electronic media device?” Volunteers who responded less than two hours reached screen time recommendations [1, 20].

#### Habitual human behaviors (sleep, SB, LPA, and MVPA)

The total night sleep time, SB, light PA (LPA) and MVPA were evaluated using a GT3X accelerometer (ActiGraph^**TM**^ Corp., Pensacola, Florida, USA) on the right side of the hip fixed by an elastic belt, 24 hours a day. Data were collected for eight consecutive days, including on the weekend. Only valid data collected for at least three nights of proper sleep were entered in the analyses, including one weekend night (Friday or Saturday) [41]. All volunteers were instructed to maintain their usual routine and to only remove the equipment to perform activities where the accelerometer would be submerged in water [42–46]. Participants were contacted daily through a messaging application during the collection period to ensure that the accelerometer was being used appropriately [47].

The collections took place over 24 hours for at least four days. Only data collected for at least 10 hours of wakefulness [47–49] and the first day of registration was excluded because it was considered a familiarization period to avoid the *Hawthorne effect* [50]. All collections were initiated at 11:59 a.m. [46].

The *Actilife*^*®*^ version 6.13.4 software program (ActiGraph, LLC, Fort Walton Beach, USA) was used to initialize, download, and process the data. A sampling rate of 30 Hz, normal filter, and 60 seconds epochs were used. The non-use time during wakefulness was defined as at least 20 consecutive minutes of zero counts/minute recording [51].

The algorithm proposed by Sadeh [52] to classify each minute as sleep or wakefulness, and the automatic algorithm validated for data collected by accelerometers used in the waist in 24 hours [47] were used in the ActiLife® software program to identify the total time of night sleep from the reintegration of the data into 60 seconds epochs.

The weekly total and average total daily sleep time were calculated only using the days when the total accumulated sleep period was ≥ 160 minutes [41]. The volunteers reached the sleep recommendations when the average total sleep time reached values between 8 and 10 hours per night [1, 18, 19].

After excluding the total time of night sleep and the non-use time in wakefulness [44], the time in SB, LPA, MVPA was classified according to the cut-off points validated for Brazilian adolescents as proposed by Romanzini et al. [53]. The volunteers reached the PA practice recommendations when they performed an average of more than 60 minutes of MVPA per day [1, 15–17].

#### Co-variables

Age and sex were self-reported. Socioeconomic status (SES) was classified through a questionnaire proposed by the Association of Research Companies [54] through the final score obtained by household characteristics, the education level of the head of the family, and the access conditions to public services.

### Statistical analysis

Descriptive statistics are presented as mean ± standard deviation, median, counts, and percentage (%). HHB daily recommendations (yes/no) were analyzed by frequency distribution and reported as percentage. A z-test (in a two-tailed hypothesis) and 95% confidence interval (CI) based on the chi-squared distribution were used for comparing two independent proportions. Univariate standard comparisons between sexes were made by Wilcoxon’s signed-rank test. Effect size is given by the biserial correlation (r) with matched pairs using 200 *bootstrap* replicates to calculate 95%CI. Effect size was interpreted according to the values |r| <0.1 “very small”; |r| 0.1 ≤ 0.3 “small”; |r| 0.3 ≤ 0.5 “moderate”; and |r| >0.5 “large” [55].

The compositional nature of 24-hour time-use behaviors (each part of the composition normalized to sum 1440 min) was analyzed according to the compositional data analysis (CoDa) paradigm using an isometric log-ratio *(ilr)* data transformation [25]. With the absence of zero values in the original dataset in any compositional part, the *ilr* coordinates were created using a sequential binary partition process (SBP) by partitioning the composition [56]. The relative dispersion of compositional data was robustly estimated using the variation matrix (i.e. contains all pair-wise log-ratio variances) [57], which summarizes the variability structure of data by log-ratio variances [25]. The multivariate outlier detection procedure was based on (robust) Mahalanobis distances in the *ilr* coordinates to reduce the effect of the deviations on the model assumptions and estimation.

The compositional regression models were subsequently used to investigate the predictive adjustments in BMI *z*-scores (*z*BMI) models. The compositional predictor (expressed as a set of *ilr* coordinates) was used as the exploratory variable. The model simplification method adopted was both directions stepwise-selected lowest Akaike information criterion (AIC) [58]. The variance inflation factor (VIF) was used as measure to analyze the multicollinearity magnitude of model terms considered satisfactory when less than 5 [59]. All other modelling assumptions (skewness, kurtosis, link function, and heteroscedasticity) were assumed [60]. The final fitted model was subsequently used for prediction purposes to quantify how hypothetical time reallocations (i.e., 15, 30, 45, 60, 75, 90, and 120 min) between each HHB were associated with BMI changes. The differences between the pivot coordinate representations of the hypothetical relocation and the average compositions of baseline HHB were (re)calculated to estimate the BMI change associated with one-to-one relocations [26]. The estimated differences for BMI and their respective 95%CIs were obtained by considering them significant when the 95%CI did not cover zero.

The analyses were performed with the *compositions* [61] and *robcompositions* [62] packages using the R statistical programming language (version 4.1.0; R Foundation for Statistical Computing, Vienna, Austria) [63]. The alpha level was set at 0.05 and residual values were adjusted (-1.96 <*z*>1.96).

## Results

**Fig 1:**
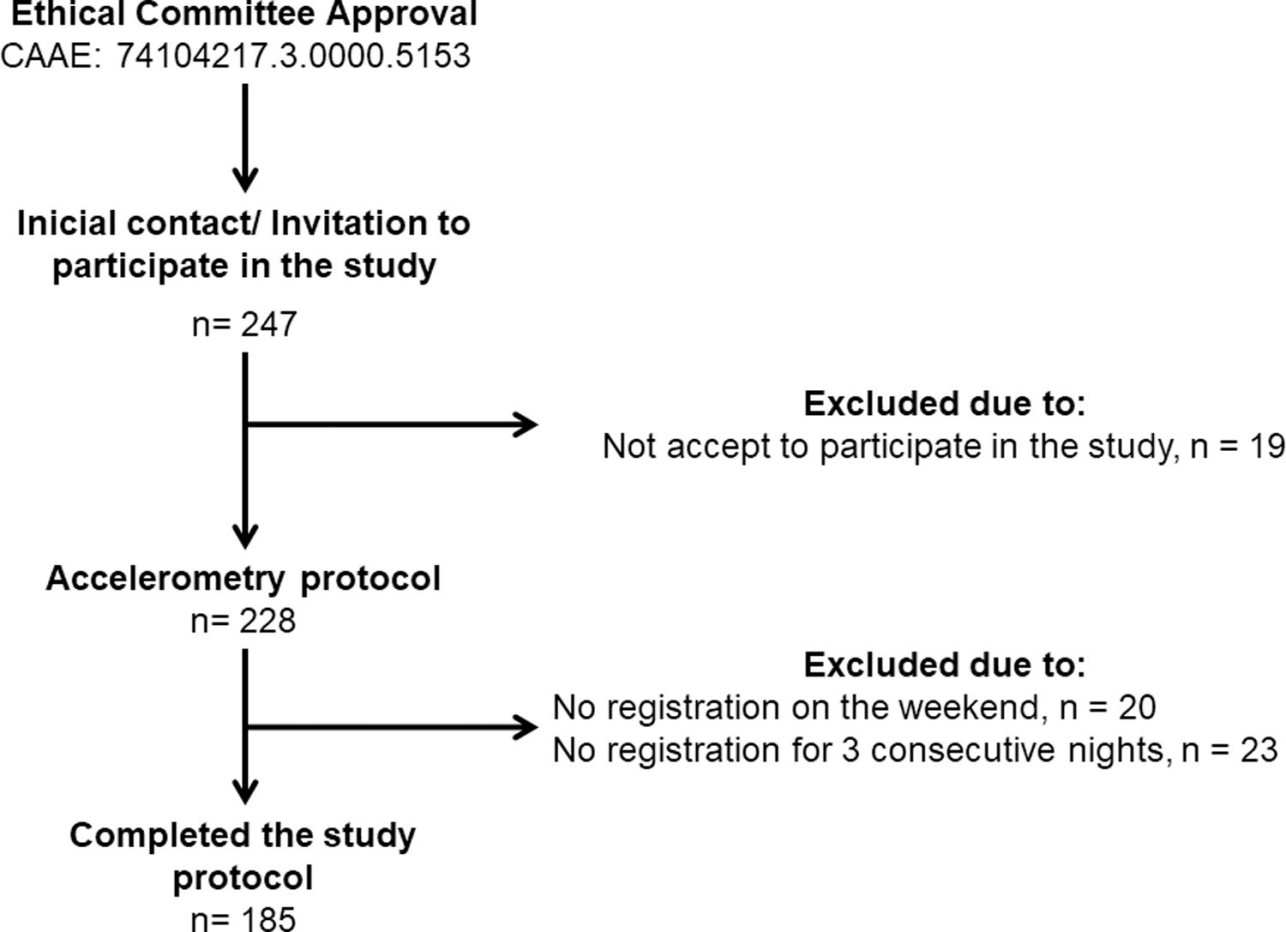
Sample selection flowchart.

A total of 247 adolescents were invited to participate in the data collection. From this total, 19 did not accept to participate in the study, and 43 were excluded for not using the accelerometer for at least three nights (two weekdays and one weekend day). Thus, the sample consisted of 185 adolescents (Fig 1).

The participants’ general characteristics are presented in Table 1. The mean age of the total sample was 15.96 ± 1.02 years, and approximately half (50.8%) of the sample was male. The participants’ characteristics were generally similar, except for the difference in physical attributes (body mass and height), in which boys are taller and heavier than girls *(both*, *p*<0.001). It is noted that both girls and boys have a high percentage of the total daily screen time exposure (27% and 30%, respectively).

**Table 1:** Participant characteristics.

Characteristics	Boys, N = 94 ^a^	Girls, N = 91 ^a^	*W-value* ^*b*^	*p-value* ^*b*^	ES (95% CI) ^c^
Age (years)	15.96 ± 1.02 (16.00)	16.13 ± 0.98 (16.00)	3843	0.214	-0.10 (-0.26, 0.07)
Weight (kg)	67.34 ± 13.60 (63.70)	59.44 ± 13.56 (56.40)	5931	<0.001	0.39 (0.24, 0.52)
Height (cm)	174.21 ± 6.61 (173.60)	162.62 ± 6.17 (162.4)0	7691	<0.001	0.80 (0.73, 0.85)
BMI (kg/m^2^)	22.17 ± 4.23 (20.98)	22.46 ± 4.99 (21.00)	4262	0.968	0.00 (-0.17, 0.16)
Socioeconomic score (a.u.)	32.91 ± 8.32 (32.00)	30.78 ± 8.46 (31.00)	4861	0.108	0.14 (-0.03, 0.30)
Sreen-time (hours)	6.42 ± 2.97 (5.00)	7.10 ± 3.60	3751	0.146	-0.12 (-0.28, 0.04)

a. u.–arbitrary unit; BMI, body mass index.

^a^ Mean ± SD (Median).

^b^ Wilcoxon’s Signed Rank Test (W-value and correspondent exact or rounded *p-value*).

^c^ Effect size (ES) was obtained by biserial correlation (r) with matched pairs (95% IC; 200 *bootstrap* replicates) and magnitude difference.

**Fig 2:**
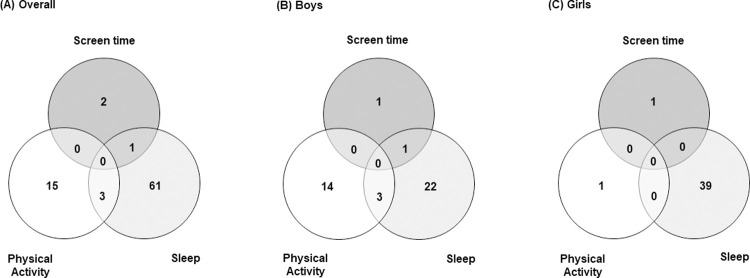
Participants’ compliance with the behavior guidelines. (A) Overall.(B) Boys. (C) iGrls.

The accelerometers were used on average for six valid days (56.2% of the sample), and the effective daily monitoring time reached 94.7 ± 3.1% per day (i.e. 1363.5 ± 45.3 min/day of the 1440 min/day possible). None of the evaluated participants, regardless of sex, simultaneously complied with all general recommendations of sleep, PA and ST in 24 h. This fact can be observed by the absence of data in the areas of union/intersection between the criteria observed in the *Venn* diagram (Fig 2A–2C). The sleep recommendation was achieved by the highest number of adolescents (N = 61; 32.97% of the total), followed by MVPA (N = 15; 8.10% of the total) and ST (N = 2; 1.08% of the total) as shown in Fig 2A. In addition, the results showed that a higher percentage of boys met the MVPA recommendations (14.89% vs. 1.09%; Z = 3.44, *p*<0.001, r = 0.84, 95% CI [0.79, 0.87]) (Fig 2B); while a higher percentage of girls meet sleep guidelines (42.85% vs. 23.40%; Z = 2.81, *p*<0.01, r = 0.82, 95%CI [0.77, 0.86]) (Fig 2C). There were no differences between sexes to meet ST recommendations (boys = 1.06%, girls = 1.09%; Z = 0.02, *p* = 0.98).

Table 2 presents the compositional statistics of the four parts of the daily time use behaviors (absolute and relative value). Standard and normalized statistics in accordance with the compositional paradigm for 1440 min/day are also presented. The normalized geometric mean for 100.0% of the time in the total sample demonstrates that an average of 34.31% of the daily time of 24 h is spent sleeping, 50.52% is destined to SB, 12.82% in LPA, and 2.35% in MVPA.

**Table 2:** Descriptive and compositional statistics of daily use of time in each human behavior.

Behaviors	Daily time (min/day)			Geometric Mean (%)		
	Overall (N = 185)	Girls (N = 91)	Boys (N = 94)	Overall (N = 185)	Girls (N = 91)	Boys (N = 94)
SB	722.13 ± 64.64	728.30 ± 58.69	716.16 ± 69.72	50.52	50.13	50.87
LPA	187.92 ± 45.94	177.05 ± 39.23	198.45 ± 49.59	12.82	13.53	12.10
MVPA	37.91 ± 18.93	31.13 ± 13.96	44.47 ± 20.78	2.35	2.79	1.96
Sleep	492.04 ± 60.13	503.53 ± 55.84	480.92 ± 62.31	34.31	33.55	35.07

SB: sedentary behavior; LPA: light physical activity; MVPA: moderate-to-vigorous physical activity. The activity behaviors in its absolute time per day and relative (%, geometric) form are presented as mean ± Standard Deviation; in the compositional perspective, the data are presented as geometric mean normalized to 100% of time (i.e. the center of compositional mean which cannot include SD) for four-part daily time-use composition (closed to total one day; i.e. 1440 min.day^-1^).

The data variability is shown in Table 3, being represented by the matrix of variation of the composition of the HHB. The minor variation of the logarithmic ratio in pairs was identified between sleep and SB (0.03), indicating greater co-dependence between these behaviors. However, the most significant variations occurred between SB and MVPA (0.34) and sleep and MVPA (0.33), constituting pairs of behaviors with the lowest co-dependence. The robust total variance of the compositional measurement (i.e. global dispersion of a compositional sample) was equivalent to 0.28.

**Table 3:** Pair-wise log-ratio matrix of the four-part time-use composition.

t _ij_	SB	LPA	MVPA	Sleep
**SB**		0.11	0.34	0.03
**LPA**			0.17	0.11
**MVPA**				0.33

SB: sedentary behavior; LPA: light physical activity; MVPA: moderate-to-vigorous physical activity. Data are presented as the variation matrix of the pair-wise log ratio estimate by robust procedures (i.e. *Minimum Covariance Determinant*, a highly robust estimator of multivariate location and scatter via the “Fast MCD” or “Deterministic MCD” ["DetMcd"] algorithm with number of subsets used for initial estimate nsamp = 500). To simplify, only the upper triangle is represented, omitting the first column and last row. A value approaching "0" indicates high proportionality between pairs of behaviors, while approaching "1" indicates the opposite.

The parameters of the fitted final multivariate linear model analyzed by ANOVA considered that isometric logarithmic coordinates and age could significantly predict *zBMI*. The model explains a moderate and statistically significant variance proportion (R^2^ = 0.10, F(4, 180) = 4.79, *p*<0.001, adj. R^2^ = 0.08). The effect of age is statistically significant and positive (β = 0.28, 95% CI [0.14, 0.42] t(180) = 3.87, *p*<0.001), confirming the need to control this effect of age in the context of isotemporal prediction analyses.

Theoretical estimates of isotemporal relocation were generated from this model using fixed time durations (15, 30, 45, 60, 75, 90, and 120 minutes) from one HHB to another, while the remaining behaviors were kept constant. The only changes predicted in the *z*BMI were observed for relocation of 75, 90, and 120 minutes of MVPA to LPA, associating with an increased perspective of 0.75, 0.76, and 0.78 units in the *z*BMI mean, respectively, as shown in Table 4.

**Table 4:** Estimated changes in the *z*BMI associated with the theoretical temporal reallocation between habitual human behaviors.

Behavior change	Isotemporal reallocations and estimated *z*BMI changes (95%CI low, high)		
	*75 minutes*	*90 minutes*	*120 minutes*
↑ **Sleep** ↓ **SB**	0.03 (-0.16, 0.22)	0.04 (-0.19, 0.26)	0.05 (-0.24, 0.35)
↑ **Sleep** ↓ **LPA**	-0.06 (-0.42, 0.30)	-0.07 (-0.53, 0.38)	-0.11 (-0.82, 0.59)
↑ **Sono** ↓ **MVPA**	0.69 (-0.15, 1.53)	0.69 (-0.15, 1.54)	0.60 (-0.16, 1.55)
↑ **SB** ↓ **Sleep**	-0.03 (-0.22, 0.17)	-0.03 (-0.27, 0.21)	-0.04 (-0.36, 0.28)
↑ **SB** ↓ **LPA**	-0.09 (-0.44, 0.27)	-0.11 (-0.56, 0.35)	-0.16 (-0.86, 0.54)
↑ **SB** ↓ **MVPA**	0.66 (-0.17, 1.49)	0.65 (-0.18, 1.49)	0.65 (-0.20, 1.49)
↑ **LPA L** ↓ **Sleep**	0.04 (-0.23, 0.31)	0.05 (-0.27, 0.36)	0.06 (-0.35, 0.47)
↑ **LPA** ↓ **SB**	0.07 (-0.18, 0.32)	0.08 (-0.21, 0.38)	0.11 (-0.27, 0.49)
↑ **LPA** ↓ **MVPA**	0.75 (0.01, 1.48)*	0.76 (0.03, 1.49)*	0.78 (0.07, 1.49)*
↑ **MVPA** ↓ **Sleep**	0.33 (-0.07. 0.73)	0.36 (-0.08, 0.81)	0.43 (-0.11, 0.96)
**MVPA** ↓ **SB**	0.36 (-0.03. 0.75)	0.40 (-0.03, 0.83)	0.48 (-0.03, 0.99)
↑ **MVPA** ↓ **LPA**	0.27 (-0.37. 0.90)	0.29 (-0.47, 1.05)	0.31 (-0.72, 1.35)

*Considering significant when the 95% CI did not cover zero.SB: sedentary behavior; LPA: light physical activity; MVPA: moderate-to-vigorous physical activity. Estimation of change in BMI *z*-scores (*z*BMI) (low, high 95% confidence interval) when the behaviors was increased (↑) or decreased (↓) time in a combination of behavioral pairs. Analysis adjusted for age after linear model adjustment of explanatory variable elimination using ordinary least squares (OLS).

## Discussion

Developing interventions to change adolescents’ behavior throughout the day is necessary. Some studies [64, 65] point to the high prevalence of children and adolescents who do not meet the guidelines for behaviors in 24 hours, presenting high ST, low MVPA, and little sleep time.

Our findings confirm this observation because none of the participants simultaneously met the guidelines for ST, MVPA, and sleep, with an average of 50.52% of the daily time of these adolescents consumed in SB (722.13±64.64 minutes), 34.31% asleep (492.04±60.13 minutes), while only 12.82% in LPA practice (187.92±45.94 minutes) and 2.35% in MVPA (37.91±.18.93 minutes). These results agree with studies conducted with Canadians aged 10 to 13 years [27], Americans aged six to 17 [33], and Czechs from eight to 18 years. Given this scenario, the risk of harmful health effects increases, in addition to impacting BMI, favoring overweight and obesity.

In our sample, 67.03% did not reach sleep time recommendations of 8 to 10 hours daily [1, 18, 19] for adolescents. This high prevalence may be linked to the fact that children’s sleep duration and quality decrease significantly as they progress from childhood to adolescence [66] due to the delay in melatonin secretion by the pineal gland, necessary for the onset of sleep [67, 68].

Consequently, most adolescents stay up late, sleeping less on the days they go to school [69], and changes in sleep patterns may be tied to physiological, behavioral, and social changes that occur during adolescence [70]. Sleep phase delay can result from changes in the operational speed of the Circadian Timing System triggered by hormonal changes during puberty. As a result, a reduction in total sleep time and an extended endogenous period longer than 24 hours is observed, leaving individuals at this stage of life with a tendency to have nocturnal habits [71].

It is essential to highlight that sleep duration influences biological processes such as inflammation, glucose regulation, appetite, and energy expenditure [72], and the reduction of total sleep time can affect appetite for decreased leptin and increased ghrelin resulting in increased hunger and food intake; all of which may favor developing obesity [73].

In addition, high screen exposure levels at night can contribute to low sleep duration [74]. The use of electronic devices that have an illuminated screen (such as televisions, laptops, tablets, smartphones, among others) [64] emits light in the blue spectrum, which increases brain and physiological arousal, in addition to attenuating melatonin release [70]. These conditions together can slow circadian rhythmic activity in teenagers, causing delayed sleep onset [63, 68]. Therefore, considering that academic activities have fixed times to take place, this may be a possible explanation for the reduction in total sleep time observed in the present study.

In our sample, 98.92% of the adolescents did not reach ST recommendations, being exposed to the screen three times longer on average than the daily recommended time, which may be tied to the high percentage of adolescents who do not reach the sleep recommendations. This picture is worrisome because the use of the Internet, and consequently social networks and Internet games for long hours can be a risk factor for digital disorders such as nomophobia, fomo and cyberchondria that lead to various psychological disorders, especially for individuals aged 12 to 18 [75]. All evaluated students remained in the school environment from 7:30 a.m. to 4:50 p.m., attending eight classes per day. Therefore, there are few possibilities to perform PA, except the daily recess moments (20 minutes in the morning and the afternoon), lunchtimes (11:10 a.m. to 1:10 p.m.), and Physical Education classes (two consecutive class schedules once a week). This daily routine favors activities performed in SB. It validates the limited prospects of time for these adolescents to be physically active, reflecting the high time allocated to SB (approximately 12 hours) and the high prevalence (90.3%) of inadequate daily recommendations of 60 minutes of MVPA [1, 15–17]. However, this panorama is not exclusive to Brazil. A recent study showed that 81% of the world’s adolescents do not meet these recommendations [76].

Regarding the compositional analysis of the day’s 24h, the behavior distribution was associated with *z*BMI with age as a predictor variable of the statistical model. The results indicate that for each year added to the sample, there is a perspective of a 0.28 unit increase in the *z*BMI mean.

This occurs due to increased growth rate, body weight, fat-free mass and mineral content during adolescence, and girls have puberty onset earlier than boys [77]. In addition, girls have a higher amount of fat mass than boys because, regardless of chronological age, pubertal development is associated with increased body fat [78]. However, boys have reduced body fat, increased shoulder length and leg-trunk length ratio, and higher growth rate peaks [77].

Regarding the isotemporal substitution of 75, 90, and 120 minutes of MVPA to LPA, a possible average increase of 0.75, 0.76, and 0.78 was found in the *z*BMI, respectively, which was also observed by replacing 30 minutes in a study conducted with New Zealand adolescents [22]. A possible justification for the results found in our study may be the fact that the sample is very homogeneous regarding BMI (68.6% eutrophic).

The World Health Organization [78] points out that PA practice of any intensity, including LPA, is fundamental in changing from SB to active, causing health benefits. Thus, LPA could act as a gateway to more intense activities. However, MVPA recommendations should be achieved for more important metabolic impacts on adolescent health to be achieved.

Therefore, maintaining or increasing the time engaged in MVPA should be the focus of interventions or programs to prevent obesity in order to avoid significant and undesirable effects on adiposity [30]. Furthermore, such strategies should mainly be adopted in adolescence, since there is a tendency to reduce PA levels as adolescents academically progress. PA is less structured and reflects on the motivation to remain physically active in adulthood [79].

It is also emphasized that LPA has been associated with some health benefits. However, it is not as effective as MVPA in preventing and treating obesity [80], especially when replacing the SB, as verified in a study conducted with boys in another Federal Institute in Brazil [81]. On the other hand, Moura et al. [81] found positive results in metabolic (HDL-C and HOMA2-S) and physiological (systolic blood pressure) indicators, showing that increased LPA practice is an effective alternative to reducing SB, in addition to the relocation of SB in favor of MVPA being associated with reduced body fat. These findings justify replacing time involved in SB and LPA for MVPA generally proposed in schools, domestic, and community environments [30].

This study’s main strength is the use of a compositional approach to evaluate behaviors in 24 hours evaluated by means of objective measurement, as well as using a cut-off point for PA and SB developed with Brazilian adolescents. In addition, it is worth noting the participants’ high adherence to the accelerometer use protocol for more than 22 hours per day, demonstrating the reliability of the data obtained.

Our study has limitations that should be considered. The main limitation is the study’s cross-sectional nature, which prevents us from making causations. In addition, evaluation of ST performed by self-report may introduce interpretation bias, and removing the accelerometer during aquatic activities (i.e. bathing and swimming) may have underestimated LPA and MVPA. The absence of muscle mass evaluation and the recording of the menstrual cycle in girls may interfere in the metabolism and sleep health. Furthermore, sleep environment characteristics (snoring, light on, noises, presence of a partner) could be used as confounding factors in the analyses if they were assessed.

## Conclusions

None of the evaluated patients reached the three recommendations (sleep, PA, ST) in 24 hours simultaneously, and few adolescents even achieved them in isolation.

A significant association was found between HHB composition and *z*BMI. The replacement of 75, 90, and 120 minutes of MVPA by LPA were associated with increased zBMI. Although this indicates a negative outlook for adolescent body composition, this would only occur over long periods (greater than 75 minutes).

The HHB relocation estimates over the day’s 24 hours did not show positive effects on *z*BMI, nor did it increase the time engaged in MVPA, which may raise the hypothesis that other parameters related to obesity as well as the interaction of these factors need to be better understood.

Further studies evaluating parameters such as eating behavior, girls’ menstrual period, and hormonal dosages which may add value to the association between the evaluated HHB and BMI are necessary. In addition, qualitative, longitudinal and intervention studies, which are essential to assess the causality between HHB and BMI, investigating the time proportion spent in different forms of LPA and SB (e.g., recreational ST, classroom time, social media exposure) and other qualitative sleep characteristics (e.g., efficiency, daytime sleepiness, insomnia, feeling tired or fatigued upon awakening) as components of adolescents’ day and their relationship with obesity would be useful.

## Supporting information

S1 File (XLSX)
